# The Yin and Yang of Alarmins in Regulation of Acute Kidney Injury

**DOI:** 10.3389/fmed.2020.00441

**Published:** 2020-08-21

**Authors:** Vikram Sabapathy, Rajkumar Venkatadri, Murat Dogan, Rahul Sharma

**Affiliations:** Division of Nephrology, Department of Medicine, Center for Immunity, Inflammation, and Regenerative Medicine (CIIR), University of Virginia, Charlottesville, VA, United States

**Keywords:** alarmins, AKI, inflammation, regeneration, IL-33, T-regulatory cells, Cytokines, DAMP

## Abstract

Acute kidney injury (AKI) is a major clinical burden affecting 20 to 50% of hospitalized and intensive care patients. Irrespective of the initiating factors, the immune system plays a major role in amplifying the disease pathogenesis with certain immune cells contributing to renal damage, whereas others offer protection and facilitate recovery. Alarmins are small molecules and proteins that include granulysins, high-mobility group box 1 protein, interleukin (IL)-1α, IL-16, IL-33, heat shock proteins, the Ca^++^ binding S100 proteins, adenosine triphosphate, and uric acid. Alarmins are mostly intracellular molecules, and their release to the extracellular milieu signals cellular stress or damage, generally leading to the recruitment of the cells of the immune system. Early studies indicated a pro-inflammatory role for the alarmins by contributing to immune-system dysregulation and worsening of AKI. However, recent developments demonstrate anti-inflammatory mechanisms of certain alarmins or alarmin-sensing receptors, which may participate in the prevention, resolution, and repair of AKI. This dual function of alarmins is intriguing and has confounded the role of alarmins in AKI. In this study, we review the contribution of various alarmins to the pathogenesis of AKI in experimental and clinical studies. We also analyze the approaches for the therapeutic utilization of alarmins for AKI.

## Introduction

Acute kidney injury (AKI) is a global problem associated with high mortality, morbidity, and clinical burden (1). AKI is defined as an abrupt deterioration of kidney function indicated by an increase in circulating levels of creatinine and blood urea nitrogen (BUN) and a decline in urine output and glomerular filtration rate (GFR) (1). Several factors can result in AKI including ischemia/reperfusion injury (IRI), sepsis, hemodynamic changes, systemic inflammation, muscle wasting, and nephrotoxicity (2, 3). The pathophysiology of AKI is multifaceted, exhibiting inflammation, tubular injury, and vascular damage (4), and can cause damages to the brain, heart, and lungs in the long run. There is no approved drug for treating AKI patients, and current clinical care involves renal replacement therapy (RRT) (1).

**Figure 1:**
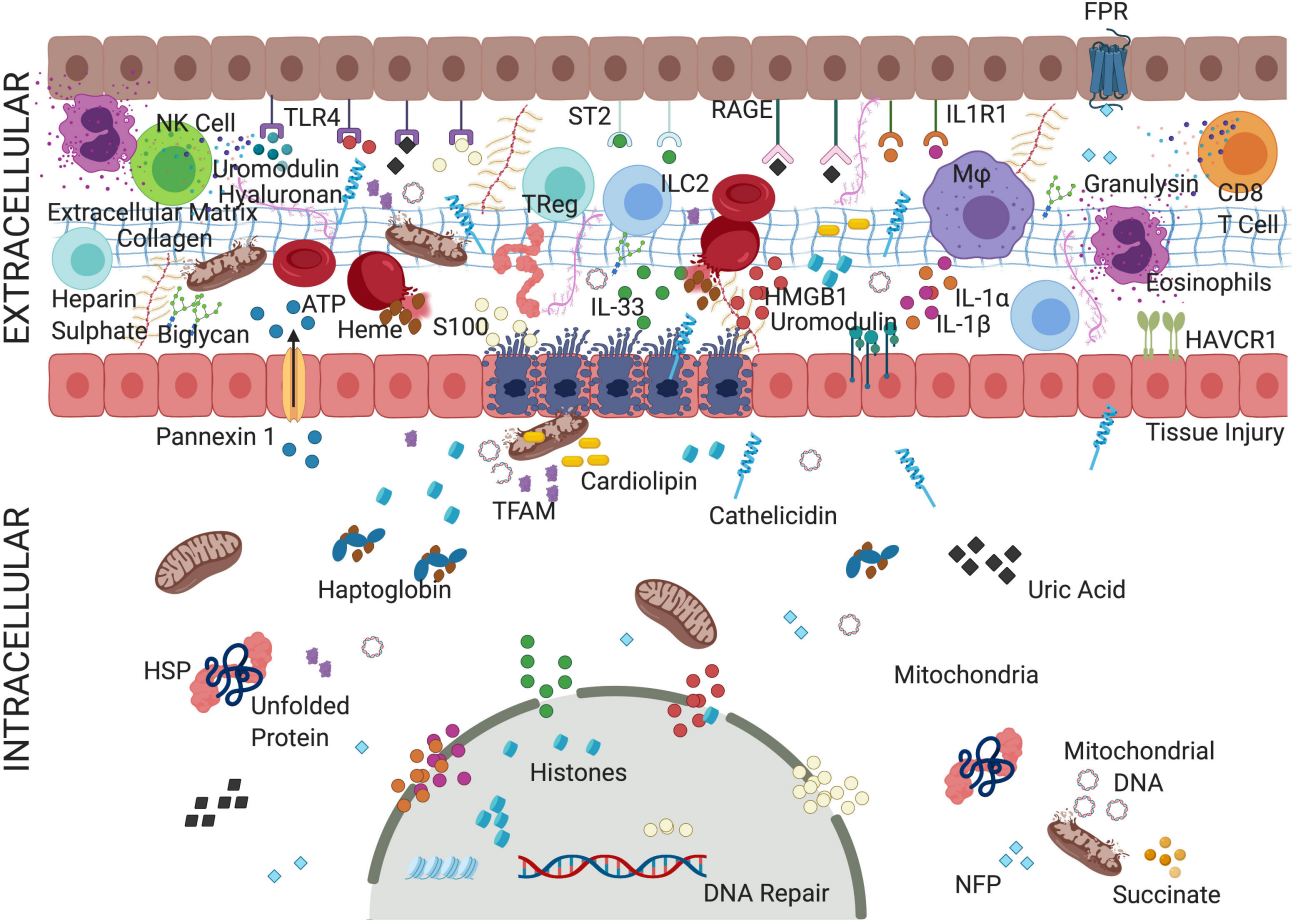
Convoluted mechanism of action of alarmins. An overview depicting complex but critical mechanisms of action of alarmins. During AKI, various biomolecules, termed alarmins, are triggered that include various proteins, non-protein small molecules, metabolites, and cellular organelles. Alarmins have been implicated in both pro-inflammatory activity, promoting inflammatory cells, and mounting anti-inflammatory effects and facilitating repair. The alarmins are classified as nuclear, cytosolic, cell membrane, extracellular matrix, and secreted (granule-derived). Most of the alarmins exert their pathological effects through cell surface receptors such as TLRs, IL-1Rs family, or RAGE triggering activation of various downstream targets such as NF-κB and interferon responsive factors (IRFs). Adenosine triphosphate (ATP), cathelicidins, defensins act through ionotropic, metabolic, and purinergic receptors, which facilitate the organization of NLRP3 inflammasome complex. Alarmins such as heparin sulfate (HS) and insulin-like growth factor–binding protein 7 (IGFBP7) binds to growth factor receptors activating immunomodulatory and prosurvival signals. Fragmented mitochondria released from the damaged cells could trigger inflammatory milieu. Thus, various alarmins activated during cellular injury not only induce inflammatory cells but act as a source of biomarkers and recruit regulatory cells to resolve the inflammation and initiate tissue repair. The specific role of alarmins in tissue injury, inflammation, and repair is underexplored but slowly evolving. For details refer to manuscript text. TLRs, Toll-like receptors; IL-1R, interleukin 1 receptor; IL-1RL1, interleukin-1 receptor-like 1 receptor; RAGE, receptor for advanced glycation end products; interleukin 33 (IL-33); interleukin 1α/β (IL-1α/β); HMGB1, high mobility group box 1; HAVcr-1, hepatitis A virus cellular receptor 1; HSPs, heat shock proteins; NFP, N-formyl peptides.

**Figure 2:**
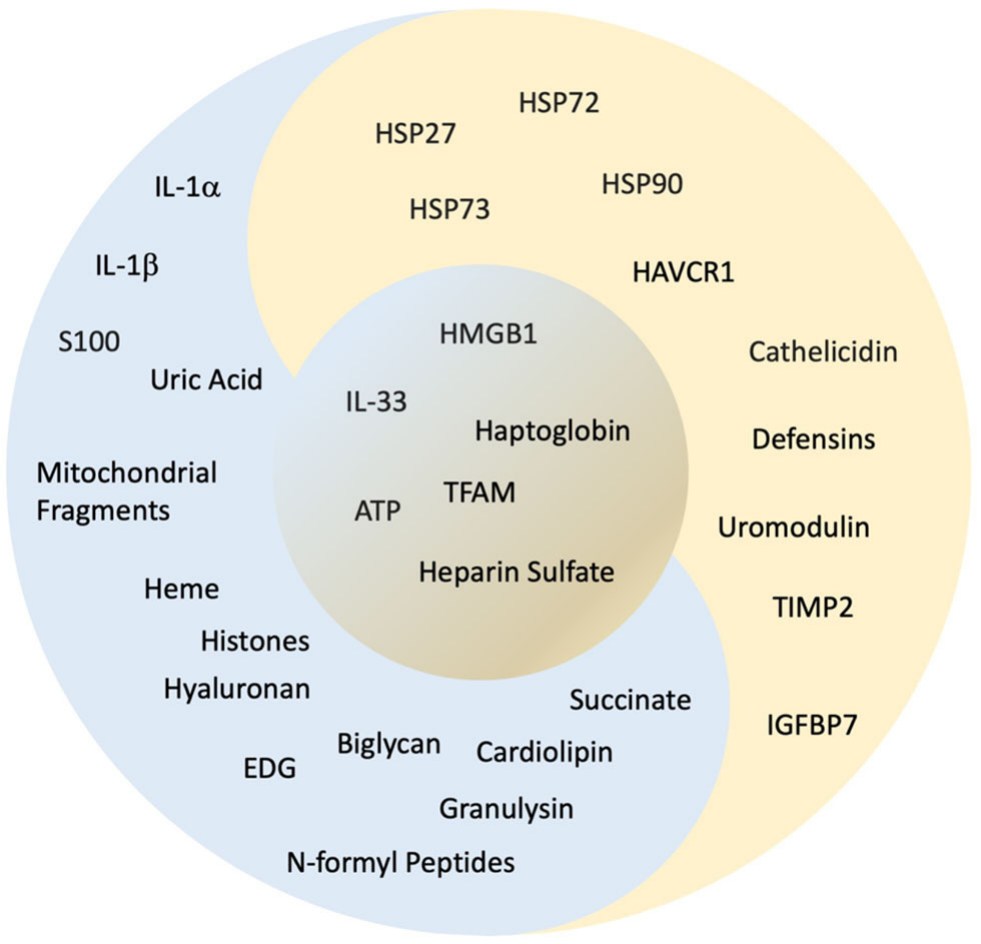
“Yin and yang” classification of alarmins. The concept of yin and yang is dualism. It shows how apparently opposing or contrary powers can really be similar, intertwined, and interdependent in the natural universe and how they can give rise to each other as they are engaged during AKI. Here, based on the available evidence, we have classified the alarmins, which have a negative influence as “yin” as represented in “blue,” alarmins with positive influence as “yang” represented in “gold” and alarmins with both “yin”/“yang” qualities are placed in the center represented in contrast between “blue” and “gold.” Refer to Figure 1 and Table 1 for abbreviations and the text for details.

With the ever-changing definitions of damage-associated molecular patterns (DAMPs) and alarmins, newer criteria were established during the International DAMP & Alarmins meeting held in Japan in November 2019 (5). “Alarmins” are a class of endogenous immunomodulatory molecules released or expressed by living cells upon cell injury, death, stress, or infection that triggers activation of the immune system (5, 6). In February 2006 in an European Molecular Biology Organization workshop on innate danger signal held in Milano, Italy, Dr. Joost Oppenheim coined the term “alarmin” to designate endogenous molecules that signal tissue and cellular damage (7). Originally proposed by Dr. Polly Matzinger, DAMPs are endogenous molecules released upon non-programmed cell death that triggers inflammatory and immune responses (8), whereas pathogen-associated molecular patterns (PAMPs) are derived from invading microbes, for example, lipopolysaccharides (LPSs) that exhibit distinct biochemical property such that they alert intrusion of the pathogens (9). The PAMPs and DAMPs were shown to trigger specific pattern recognition receptors (PRRs), for example, Toll-like receptors (TLRs) for immune activation (10, 11). Although DAMPs may now be recognized as molecules that are released or secreted from dead cells, and alarmins constitute molecules that are secreted by living cells (5), there is still a lot of overlap and ambiguity in the literature. Nevertheless, to our understanding and for the purpose of this review, all DAMPs are alarmins, but not all alarmins are DAMPs. Several types of alarmins have now been recognized and are classified as nuclear, cytosolic, mitochondrial, extracellular matrix, and secreted (granule-derived) (Table 1). Recent evidences suggest that alarmins are pleiotropic factors that promote both inflammatory and regulatory responses (6). Both alarmins and their receptors are emerging as important biomarkers in a variety of disease conditions (6). Here, we review and discuss the inflammatory, regulatory, and regenerative capabilities of alarmin as it relates to AKI (Figure 1). Based on the available literary evidence, we classify the “yin” and “yang” of alarmins (Figure 2).

**Table 1:** Classification of alarmins.

**Origin**	**Types**	**Receptors**	**Preclinical**	**Clinical**
Nuclear	HMGB1	CXCR4, RAGE, TLR2,4,9 (12)	(13)	(14)
	IL-1α	IL-1R (15)	(15)	(16)
	IL-33	IL-1RL1 (ST2) (17)	(17, 18)	(19)
	Histones	TLR2,4 (20)	(20)	—
Cytosolic	Heat shock proteins	TLR2,4, CD91 (21)	(22)	(23)
	S100 proteins	RAGE, TLR4 (24)	(24)	(25)
	Uric acid	P2X7 (26)	(27)	(28, 29)
	Haptoglobin	CD163 (30)	(31)	(32)
	Heme	TLR4, CD91, CD163 (33)	(34)	(35)
Mitochondrial	Mitochondrial fragments	—	(36)	(37)
	ATP	P1, P2Y2,6,12, P2X1,3,7 (38)	(39)	(40)
	Mitochondrial DNA	cGAS, endosomal TLR9, AIM2, NLRP3 (41)	(42)	(42–44)
	N-formyl peptides	FPR (45)	(46)	(45)
	TFAM	—	(47, 48)	—
	Succinate	GPR91 (49)	(50)	(51)
	Cardiolipin	CD1d (52), NLRP3 (53)	(54)	—
Cell membrane	HAVCR1	—	(55)	(56–58)
	Uromodulin	TLR4	(59)	(60, 61)
Extracellular matrix	Heparin sulfate	FGFRs (62)	(62)	(63)
	Hyaluronan	TLR2,4, NLRP3 (64)	(65)	(66)
	Biglycan	TLR2,4 (67)	(68)	(69)
Secreted/granule-derived	Defensins (α,β)	TLR4, CCR6 (70)	(70)	(71)
	Cathelicidin (LL37/CRAMP)	TLR7,8,9, FPRL1, FPR2, P2X7 (72)	(72)	(72)
	EDG	TLR2 (73)	(74)	(73)
	Granulysin	TLR4 (75)	—	(76, 77)
	TIMP-2	MT1-MMP, integrins, AGTR2 (78)	(78)	(79)
	IGFBP7	IGF1R (80)	(78)	(79)
	TSLP	TSLPR-IL-7Rα (81)	(81)	(81)

*This table represents the majority of alarmins and DAMPs that are reportedly involved in AKI for the purpose of this review. For a more extensive understanding of DAMPS outside of AKI purview, refer to Gong et al. (11). AIM2, absent in melanoma 2; ATP, adenosine triphosphate; AGTR2, angiotensin II receptor type 2; CCR6, C-C motif chemokine receptor 6; CXCR4, C-X-C motif chemokine receptor 4; CRAMP, cathelicidin-related antimicrobial peptide; cGAS, cyclic GMP-AMP synthase; EDG, eosinophil-derived granules; FGFRs, fibroblast growth factor receptors; FPR, formyl peptide receptor; FPRL1, formyl peptide receptor like 1; GPR91, G protein–coupled receptor 91; HAVcr-1, hepatitis A virus cellular receptor 1; HMGB1, high mobility group box 1; IGF1R, insulin-like growth factor 1 receptor; IGFBP7, insulin-like growth factor–binding protein 7; IL-1α, interleukin 1α; IL-33, interleukin 33; IL-1R, interleukin 1 receptor; IL-1RL1, interleukin 1 receptor like 1 receptor; MT1-MMP, membrane type 1-matrix metalloproteinase; TFAM, mitochondrial transcription factor A; NFP, N-formyl peptides; NLRP3, NOD-, LRR-, and pyrin domain-containing protein 3; RAGE, receptor for advanced glycation end-products; TSLP, thymic stromal lymphopoietin; TSLPR, thymic stromal lymphopoietin receptor; TIMP2, TIMP metallopeptidase inhibitor 2; TLR, Toll-like receptor*.

## Pro-Inflammatory Role of Alarmins in AKI

### Nuclear Alarmins

***IL-1 family cytokines*** consisting of IL-1α, IL-1β, IL-18, IL-33, IL-36α, IL-36β, IL-36γ, IL-36Rα, IL-37, IL-38, and IL1Ra are nuclear proteins that are produced as pro-proteins and are matured by proteases (82). Interleukin 1α and IL-1β promote pro-inflammatory cytokine production by multiple immune cells in toxin-induced AKI (83). Interleukin 1α-deficient mice were protected from cisplatin-induced AKI (15). However, there was no difference in inflammatory cell infiltration between wild-type and IL-1α^−/−^ mice. The IL-1 family cytokine IL-33 has emerged as a critical factor in controlling the type 1 cytokine production. IL-33 is a nuclear protein that is typically released from the damaged cell and promotes inflammatory response (84). Increased expression of IL-33 was observed in kidneys of cisplatin and IRI-induced AKI models (17, 18). In the IRI model, IL-33 was postulated to amplify the recruitment of myeloid cells through secretion of chemokines monocyte chemoattractant protein 1 (MCP-1) and macrophage inflammatory protein 2 by the epithelial cells early after injury and promoted activation of invariant natural killer (NK) T cells in later stages (18). Following renal transplantation in patients, increased levels of IL-33 were observed in serum and urine and may contribute to renal IRI (19).

***High mobility group box 1 (HMGB1)*** is a nuclear protein that acts as a cotranscription factor and plays an important role in DNA repair, differentiation, and development (12). Upon release from the damaged cells, HMGB1 plays an active role in pro-inflammatory responses. HMGB1 exerts its pathogenic effects on kidneys through receptor for advanced glycation end products (RAGE) and TLRs including TLR2/TLR4/TLR5/TLR9 (12, 85). A cross-section clinical study demonstrated a rise in serum HMGB1 levels in patients with AKI (14). In experimental studies too, the administration of rHMGB1 after IRI exacerbated injury (13). Sepsis-induced AKI in mice with chronic kidney disease (CKD) increased the expression of vascular endothelial growth factor (VEGF) and HMGB1 levels; however, inhibition of HMGB1, but not VEGF, was found to be protective (86).

Mice with a deficiency in TLR4, one of the receptors for HMGB1, were protected against kidney IRI. Moreover, neither the anti-HMGB1 antibody nor rHMGB1 administration affected the renoprotection in TLR4^−/−^ mice (13). The results indicate that HMGB1 might promote kidney injury through TLR4 signaling. Glycyrrhizic acid could also attenuate renal IRI by inhibiting the interactions of HMGB1 with tubular epithelial cells (TECs) (87). Treatment with mycophenolate mofetil (MMF), a commonly used immunosuppressant, resulted in the improvement of renal function in IRI along with reduced levels of plasma creatinine and cytokines, as well as lower TLR4 expression (88). However, there was no change in HMGB1 levels, thus implying that MMF reduces TLR4 expression directly. Interestingly, pretreatment with carbon monoxide-releasing molecule-2 prevented the nuclear histone acetyltransferase activity by inhibiting HMGB1 release (89). This resulted in a reduction in the pathological damage to the kidney and was accompanied by downregulation of TLR4, RAGE, tumor necrosis factor α (TNF-α), IL-1β, IL-6, and MCP-1 and protection from IRI, indicating HMGB1 as one of the mechanisms of MMF treatment. Elevated levels of circulating HMGB1 were found in patients with AKI (14) and were independently associated with leukocyte count and correlated negatively with proteinuria in AKI settings.

***Histones*** are highly basic proteins, rich in arginine and lysine, and highly conserved across species. They provide structural stability to chromatin and regulate gene expression (90). Histones in extracellular space may appear either due to release from damaged cells, by pro-inflammatory cells through active secretion, or as a component of neutrophil extracellular traps from infiltrating neutrophils (91). Extracellular histones released from dying tubular cells were associated with AKI, and were found not only to exhibit direct toxicity to renal cells but to induce pro-inflammatory cytokine and activate the innate immune response in a TLR2/TLR4-dependent manner (20).

### Cytosolic Alarmins

***Heat shock proteins (HSPs)*** play an important role in a variety of cellular processes such as cryoprotection, intracellular assembly, protein folding, and translocation of oligomeric proteins (23). AKI increases the expression of HSP27, HSP72, and HSP73 in kidney tissues (21, 92–94). HSP27, HSP72, and HSP73 prevent apoptosis by decreasing intracellular reactive oxygen species (ROS) and by targeting mitochondrial caspase-dependent apoptotic pathways (92, 93, 95). They may also help with the stabilization and refolding of aggregated cellular proteins in an adenosine triphosphate (ATP)–dependent fashion (93). HSP90, on the other hand, participates in regeneration and differentiation of injured tubules (96). In a clinical study, the urinary level of HSP72 did not increase significantly in kidney transplant recipients with prerenal AKI, and a small increase in HSP70 level was noted at patients with other factors of AKI, namely, obstructive uropathy, calcineurin inhibitor drug toxicity, recurrence of primary glomerular disease, and non-steroidal anti-inflammatory drug use (97). Additionally, in the pediatric patient group, it was shown that HSP60 could be used as a diagnostic tool for AKI secondary to septic shock (98).

***S100 proteins*** are a family of cytosolic calcium-binding proteins of ~25 known members that are involved in controlling apoptosis, proliferation, differentiation, migration, energy metabolism, calcium balance, protein phosphorylation, and inflammation (99). S100A8 and S100A9 are secretory proteins that can form both heterodimers and homodimers. S100A8/A9 derived from neutrophils and monocytes acts as an activator of the innate immune system through TLR4 (24). Based on the observations that levels of S100A8/A9 were proportionally elevated with increasing severity of experimental kidney injury (24), their serum levels were utilized as an early prognostic marker of AKI associated with cardiac surgery in a clinical study (25).

***Uric acid*** crystallization has long been associated with gouty arthritis and kidney stones. However, a strong correlation of serum uric acid and AKI is emerging with multiple chronic conditions including hypertension, CKD, cardiovascular diseases, stroke, diabetic nephropathy, and metabolic syndrome (26). Uric acid acts as an antioxidant in the extracellular environment but exhibits pro-oxidant activity in the intracellular environment (100). Hyperuricemia in AKI results in dilatation of the collecting ducts leading to flattening of the epithelium, and multiple downstream consequences that include intraluminal crystal precipitation, increased intraluminal hydrostatic pressures, decrease in GFR and renal plasma flow, activation of inflammasome and necroptosis, crystal adhesion, granuloma formation, interstitial inflammation, and tubular cell injury (101, 102).

***Haptoglobin*** is a protein produced exclusively in the liver that can bind to hemoglobulin and myoglobulin (103). Interestingly, it was observed that renal cells start expressing haptoglobin in AKI (31). Paradoxically, haptoglobin was reported to participate in both pro-inflammatory and anti-inflammatory responses. On the one hand, haptoglobin could prevent respiratory burst in stimulated neutrophils, blunt endotoxin-stimulated T-lymphocyte proliferation, and modulate macrophage and dendritic cell function; on the other hand, it could also activate TLR signaling and contribute to inflammation. Furthermore, haptoglobin abruptly released from kidneys could also exert adverse pathophysiological effects in acute transplant rejection, which is also caused by AKI (30). An increase in haptoglobin levels in cardiac surgery patients has been associated with postoperative AKI indicating a direct role in ischemic AKI (32).

***Heme*** is an iron-containing, tetrapyrrole ring that is an essential prosthetic group in an array of proteins and influences cellular and metabolic functions (33). Free heme at higher than physiological levels can be cytotoxic because of its bioreactivity and pro-oxidative effects. Higher levels of heme were observed following ischemia-induced AKI (104). Mechanistically, heme contributed toward cellular toxicity by oxidizing lipids, denaturing proteins, cytoskeletal rearrangement, inhibiting enzyme activity, denaturing DNA, and affecting mitochondrial metabolism (105). It also induced pro-inflammatory response by inducing chemokines such as MCP-1 by the action of nuclear factor κB (NF-κB) signaling, increased leukocyte recruitment, and vascular permeability (34). Pigment nephropathy due to rhabdomyolysis and hemolysis accounts for ~10% of all cases of AKI (35).

### Mitochondrial Alarmins

Considered to be the powerhouse of the cell and critical for maintaining the cellular functions, mitochondria are also a source of factors that can induce cell apoptosis (106). Fragmentation of mitochondria is an important early event in the manifestation of AKI of both chemical and ischemic etiology (36). The release of cytochrome C from mitochondria into the cytoplasm is an endogenous signal for the cell to undergo apoptosis. Swollen mitochondria were observed in renal tissues in mice treated with LPS, a model of sepsis-associated AKI (107). These mitochondria stained poorly for cytochrome c oxidase, an indication of underlying reduced electron transport chain activity. The mitochondria are fast becoming a critical target, and mitochondrial DAMPs that include mitochondrial DNA (mtDNA), ATP, N-formyl peptides, TFAM, succinate, and cardiolipin [reviewed in (108, 109)] are also being identified for their pathological roles in renal injury and dysfunction as discussed below.

***Mitochondrial DNA (mtDNA)***, identified as a DAMP, has been suggested to also act as an alarmin that upon release into the cytoplasm triggers an inflammatory response and has been proposed to be used as a potential biomarker for kidney injury (109, 110). Cellular stress results in leakage of mtDNA leading to inflammation, likely through recognition by four innate receptors: cytosolic cyclic GMP-AMP synthase (cGAS), endosomal TLR9, and two inflammasomes: absent in melanoma 2 (AIM2), and NOD, LRR, and pyrin domain-containing protein 3 (NLRP3) (41). Levels of urinary mtDNA were elevated in mice after IRI-induced AKI (42). Clinical studies have indicated an association of urinary mtDNA with initiation and progression of AKI in the surgical intensive care unit (43), cardiac surgery (42), and sepsis (44) patients.

*Adenosine triphosphate (ATP)* is the vital source of energy for cellular processes, and its intracellular level is regulated by mitochondrial oxidative phosphorylation. However, extracellular ATP is an indication of mechanical stress and cellular damage (38). Binding of extracellular ATP activates the membrane-anchored ionotropic P2X (P2XRs) and metabolic P2Y (P2YRs) purinergic receptors. Activation of these purinergic signals by ATP triggers a variety of biological responses such as inflammation, tissue damage, and cell proliferation in renal diseases (38). Inhibition of purinergic receptors was protective in both ischemic and sepsis-induced AKI (111, 112). ATP and selective agonists of the P2X_7_ receptor were shown to activate peptidyl arginine deaminase 4 (PAD4) in proximal tubular cells (PTCs) and exacerbate IRI (113). Recently, the P2X_4_ receptor was shown to exacerbate ischemic AKI through NLRP3 inflammasome signaling in the renal proximal convoluted tubules (PCTs) (114). CD39 and CD73 are two ectonucleotidases that break down ATP to adenosine, which has anti-inflammatory properties (115). The absence of CD73 in mice exacerbated inflammation and worsened AKI outcomes (116), whereas mice transgenic for overexpression of human CD39 were protected against AKI (117). The release of ATP to the extracellular milieu and its intracellular levels is also regulated by pannexin receptors (39). Panx1 was recently shown to induce ferroptosis in renal IRI and its deletion protected from IRI (118, 119).

***Mitochondrial N-formyl peptides (FMIT, mtFPs, NFP, or mitocryptides)*** are similar to bacterial DAMP peptides. The evidence of the role of FMIT leading to AKI is rather indirect through the progression of sepsis. It was reported that mitochondrial N-formyl peptides induce sepsis-like syndrome, which could further affect organs including kidneys, lungs, and brain (46). It is known that a significant proportion of trauma patients presents sepsis-like syndrome without bacterial infections, and this condition is termed systemic inflammatory response syndrome (SIRS). One of the most common complications of SIRS is AKI, which is triggered by FMIT through formyl peptide receptor activation leading to hypotension and vascular collapse (45).

***Mitochondrial transcription factor A (TFAM)*** is a member of a high mobility group (HMG) box proteins (109). It is an important regulator of the transcription and replication of mtDNA, as well as a key regulator of mitochondrial dynamics and function (47). The development of TFAM-deficient mice has enhanced our understanding of the role of TFAM in renal injury. It was recently reported using this versatile mouse model that mitochondrial damage activates the widely investigated cGAS-STING pathway leading to renal inflammation and fibrosis (47). The role of mitochondrial damage and the cGAS-STING pathway was also recapitulated recently in the cisplatin-induced AKI mouse model (48).

***Succinate*** is an intermediate of the tricarboxylic acid cycle, which reaches extracellular milieu upon injury or ischemic conditions in the tissue (109). Succinate receptor GPR91 expressed in immature DCs and macrophages binds to the extracellular succinate and gets activated, resulting in either initiation or exacerbation of immune response (49). Plasma succinate levels were shown to be upregulated in studies on the changes in the metabolic profiles in murine AKI (50).

***Cardiolipin*** is a class of phospholipids that account for ~20% of lipids in the inner mitochondrial membrane (120). It is critical for many mitochondrial processes such as protein import, dynamics, respiratory chain functionally, and metabolism. Extracellular cardiolipin release due to mitochondrial stress or injury is sensed by T cells through the presentation on the major histocompatibility complex–like molecule CD1d (52). Cardiolipin can also bind to NLRP3 directly, eliciting, and inflammasome-mediated immune response (53). Peroxidation and loss of cardiolipin have been shown to contribute to pathogenesis in experimental AKI (54).

### Extracellular Matrix Associated Alarmins

The epithelial injury and inflammation in AKI also lead to disruption of the glycocalyx, an endothelial surface layer consisting of lectin and proteoglycan (62).

***Heparin sulfate (HS)*** is a major component of glycocalyx that helps in the organization of ~50% of the glycocalyx. Heparanase is an endoglycosidase enzyme that functions to cleave HS. Increased expression of heparanase has been observed in AKI, suggesting it could be used as an early biomarker (62). Shedding of glycocalyx is accompanied by reduction of endothelial nitric oxide synthase and an increase in inflammation (121). Activation of heparanase was also observed early in the sepsis-induced AKI in mice and correlated with higher pro-inflammatory cytokine levels (122). Detectability of heparanase in the urine also supported its potential as an important biomarker in sepsis–AKI (63). Further, inhibitors of heparanase activation attenuated the renal transcription of the pro-inflammatory mediators (122).

***Hyaluronic acid (HA)*** is also an important component of the extracellular matrix. It is mainly composed of N-acetyl glucosamine and glucuronic acid (64). HA synthesis has been shown to increase during fibrosis and inflammatory conditions. Endothelial cells and TECs express abnormally high levels of CD44 and HA receptor during AKI (64). Further, the uptake of HA by these cells resulted in cellular dysfunction. In a pioneering study, urinary HA was correlated with AKI in patients, also suggesting that it could be used as a biomarker to differentiate AKI from CKD in patients. Additionally, an increase in HA has been attributed to T-cell and macrophage infiltration and formation fibrosis in AKI (65).

***Biglycan*** is expressed as a component of ECM in all organs and belongs to the small leucine-rich proteoglycan (SLRP) family that is released from the extracellular matrix (68). Overexpression of biglycan is a common clinical feature in many renal pathologies. Overexpressing biglycan triggered activation of TLR2 and TLR4 to exacerbate pathophysiology of experimental AKI (67). More recently, it was reported that biglycan activates autophagy in macrophages through a novel CD44–TLR4 signaling axis in the setting of IRI (123). Both preclinical and human studies have identified soluble biglycan as biomarkers in inflammatory renal diseases [detailed specific review in (69)].

### Cell Membrane–Bound Alarmins

***Hepatitis A virus cellular receptor 1 (HAVCR1)***, initially identified as a receptor for several viruses, is also known as T-cell immunoglobulin and mucin domain 1 (TIM-1) or kidney injury molecule 1 (KIM-1). KIM-1, although expressed in multiple tissues, is not expressed in normal kidneys; however, it gets rapidly upregulated in PCT of the kidney in AKI (55). KIM-1 was the first non-myeloid phosphatidylserine receptor identified that could transform epithelial cells into “semiprofessional” phagocytes; thus, playing a role in the removal of apoptotic cells and necrotic tissue fragments (124). Recently, KIM-1 has also been attributed to the resolution of kidney inflammation, suggesting additional possible roles for this alarmin molecule or receptor (55). KIM-1 was shown to activate the ERK/MAPK signaling to promote the migration and proliferation of renal TECs (125). KIM-1 is detected in the urine of kidney injury patients and is being evaluated as a prominent biomarkers for AKI [extensively reviewed in (56–58)].

***Uromodulin or Tamm–Horsfall protein (THP)*** is a glycoprotein expressed in the thick ascending limb of the kidney and is the highest excreted protein in the urine following proteolytic cleavage (60). Although the function of uromodulin is not completely understood, it is proposed as a biomarker of kidney injury (60), polycystic kidney disease (126), and acute transplant rejection (127). Uromodulin was shown to promote immune cell activation via activating TLR4 in experimental studies (128). Clinical studies suggested that uromodulin may also be involved in the progression of CKD with its serum levels positively correlating with serum levels of pro-inflammatory cytokines (129). Paradoxically, uromodulin also has a protective effect in AKI. Uromodulin was shown to exhibit anti-inflammatory effects through reducing TLR4 expression in the thick ascending limb as kidneys from THP-deficient mice exhibited more inflammation and injury in the outer medulla (59). In cardiac surgery–associated AKI, a lower uromodulin-to-creatinine ratio correlated with higher odds of AKI and higher peak serum creatinine levels (130). In another clinical study in acute pancreatitis related AKI, serum uromodulin concentration had a positive correlation with GFR, and patients with AKI had lower serum uromodulin (131). Lower serum uromodulin levels were thus predictors of AKI in pediatric cardiac surgery (132), patients with cirrhosis (61), or renal cancer patients with partial nephrectomy (133).

### Secreted/Granule-Derived Alarmins

Many granule-derived alarmins were initially identified as antimicrobial products secreted by cells, but their role in sterile inflammation is now increasingly recognized (134).

***Defensins*** are a class of antimicrobial peptides, present in the granules of many cell types, and have a broad range of antimicrobial activity in both Gram-negative and Gram-positive bacteria (135). Defensins can be categorized into two families, the α-defensins and β-defensins (136). Although Paneth cells in the intestine are the main source of α-defensins in mice, higher levels of defensins were observed in the kidneys in glomerulonephritis and CKD (137). Elevated levels of defensin were detected after AKI and were shown to induce inflammation, injury, and impaired barrier functions in the gut (70). As a result, the delivery of defensins and other pro-inflammatory molecules such as IL-17A from intestinal macrophages to the liver resulted in hepatic inflammation and apoptosis. In turn, overproduction of hepatic IL-6 and TNF-α led to systemic inflammation and enhancement of renal dysfunction in a feed-forward loop (70, 138). Urinary β-defensins were proposed to be a useful biomarker in early prediction of contrast-induced nephropathy, which accounts for ~10 to 15% of hospital-acquired AKI (71).

***Cathelicidins*** are a family of antimicrobial and immunomodulatory peptides expressed in epithelial and immune cells under homeostasis and inflammation (139). A single cathelicidin is found in humans—hcAP18/LL-37 and rodents—cathelicidin-related antimicrobial peptide (CRAMP) (140). Cathelicidin expression was significantly downregulated in clinical AKI as well as in murine models (72). NLRP3 overactivation was discovered to be one of the major effects of this deficiency in cathelicidin that causes elevated inflammatory responses and apoptosis (141).

***Tissue inhibitor of metalloproteinases 2 (TIMP-2) and insulin-like growth*** ***factor-binding protein 7 (IGFBP7)*** have gained recognition as clinical biomarkers of AKI, collectively known as Nephrocheck™ commercially (79). TIMP-2 is a natural inhibitor of matrix metalloproteinases involved in the degradation of the extracellular matrix (142). Under steady state, TIMP-2 is expressed in monocytes, B cells, and T cells (142). Increased levels of TIMP-2 were detected in urine immediately following AKI (78). In the normal kidneys, TIMP-2 is localized in PCT. However, there was an apparent reduction of TIMP-2 signals after AKI and directly correlated to the severity of AKI (78). IGFBP-7 binds to the IGF and regulates its bioavailability in body fluids and tissues. Following AKI, a massive increase in IGFBP7 in urine was observed (78). Similar to TIMP-2 strong cortical proximal tubular staining of IGFBP7 was observed in normal under normal conditions. However, upon AKI, there was a severe reduction of proximal tubular IGFBP7 (143). Insulin-like growth factor–binding protein has been hypothesized to be involved in cellular senescence (78) and immune cell function (80). More detailed mechanistic studies are required to uncover the molecular and cellular basis of IGFBP7 in the context of inflammation.

***Thymic stromal lymphopoietin (TSLP)*** is mainly produced from stromal and epithelial cells, and its function to promote T helper type2 (TH2) cell response has linked it to allergic inflammation (144). The TSLP levels were elevated in sepsis-associated AKI in both humans and rodent models (81). TSLP was associated with NF-κB signaling to elicit the inflammatory response. Other granule-derived peptides such as those produced by eosinophils (73), and granulysins that are secreted by cytotoxic T lymphocytes and NK cells (145), were reported in renal allograft rejection (76, 77), and may also be linked with AKI and mortality (75).

## Potential Therapeutic Application of Targeting Alarmin signaling

Alarmins were initially identified as acute-phase molecules that cause immune activation and were deemed pro-inflammatory. Consequently, several approaches to inhibit alarmins and their receptors have been explored for intervention in AKI. Interestingly, several alarmins also have dual functions and can promote protective pathways and thus are being explored for therapeutic use. We review these two opposing approaches below in the context of AKI.

### Inhibiting Alarmin Signaling

#### Nuclear Alarmins

Administration of the soluble form of IL-33 receptor ST2 (sST2) was shown to prevent the onset of acute inflammation (84). It is believed that sST2 may act as a decoy receptor and neutralizes the IL-33 activity. Treatment with sST2 in the cisplatin-induced AKI model exhibited fewer CD4-infiltrating T cells, lower serum creatinine, and decreased acute tubular necrosis (ATN) and apoptosis as compared to the untreated controls (17). In contrast, treatment with recombinant IL-33 (rIL-33) exacerbated the AKI with an increase in CD4 T-cell infiltration, serum creatinine, ATN, and apoptosis (17). Interestingly, it was observed that the administration of rIL-33 did not exacerbate AKI in CD4-deficient mice, suggesting a direct effect of IL-33 activity on CD4 T cells (17). These data indicated that inhibiting the IL-33 signaling has therapeutic potential in treating or preventing AKI. Similarly, treatment with HMGB1 neutralizing antibody after IRI led to attenuation of TNF-α and MCP-1 levels and protected against kidney IRI, as evidenced by lower levels of serum creatinine, tubulointerstitial neutrophil infiltration, and tubular damage compared to the control mice (13). Various IL-1β/IL-1α/IL-1R1–specific inhibitory molecules are currently in different phases of clinical trials (16). Neutralization of histones using targeted neutralizing antibody also led to the attenuating pathogenic effect of histones, thus preventing AKI (20).

#### Cytosolic Alarmins

HSP90 transduces signals via binding to the transforming growth factor β type I (TGFβI) and type II (TGFβII) receptors (22). Blocking the interaction of HSP90 with TGFβII receptor by using 17-allyamino-17-demethoxygeldanamycin reduced fibrosis by promoting the ubiquitination of TGFβII. S100A8/A9–TLR4–NLRP3 inflammasome pathway was shown to trigger inflammation, apoptosis, and tissue injury during AKI. Inhibition of this pathway through siRNA to TLR4–NLRP3 ameliorated the kidney function in contrast-induced acute kidney injury model (24). Inhibition of TSLP, a TH2-inducing cytokine, with siRNA also resulted in lowering the sepsis-associated organ dysfunction and inflammatory cytokine levels (81).

In a rat model of cisplatin-induced AKI, moderate hyperuricemia was associated with the absence of intrarenal crystals but correlated with greater tubular injury, significant macrophage infiltration, and increased expression of MCP-1 (27). Treatment with rasburicase, a uric acid oxidase, reversed the inflammation and tubular injury (28). Many clinical approaches employed in AKI, including allopurinol, febuxostat, and Renal Replacement Therapy (RRT), may act by decreasing circulating urate to reduce its pro-inflammatory effects (29).

#### Mitochondrial Alarmins

Mitochondrial fragmentation has been thought to be one of the possible mechanisms contributing to injury in AKI. Inhibition of mitochondrial fragments was observed by blocking fission protein Drp1 along with the reduction in cytochrome c release and apoptosis (36). Similar results were obtained by blocking Drp1 using a new pharmacological inhibitor mdivi-1 (36). Targeting mitochondria by promoting mitochondrial health for therapeutic effects on AKI includes promoting metabolism by augmenting fatty acid oxidation using peroxisome proliferator-activated receptor α (PPARα) overexpression (146) or augmenting ETC using CoQ10 (ubiquinone) (147). Mitochondrial fragmentation induces ROS, which was targeted using MitoQ and SS-31 to attenuate AKI (148). Cyclosporine that is used in transplantation may also counter AKI by regulating mitochondrial membrane permeability by inhibiting cyclophilin D (149). Agents such as temsirolimus (150) function by targeting mitophagy through activating mTOR signaling. Finally, improving mitochondrial biogenesis by enhancing nuclear transcription of mitochondrial proteins using PPARγ-coactivator-1α (PGC1α) (107) or by activating β-adrenergic receptors using formoterol (151) may also contribute to protection from AKI by reducing mitochondrial fragmentation. Compound SS-31, which reenergizes mitochondria by preventing matrix swelling and preserving cristae structure, thus restoring ATP, is being clinically tested. SS-31 selectively binds to cardiolipin, preventing its peroxidation and loss (37).

Depletion of extracellular ATP with apyrase, or blocking of P2XR with pyridoxal phosphate-6-azophenyl-2′,4′-disulfonic acid (PPADS), has been shown to prevent necrosis-related inflammation (152). In the same study, treatment with A438079, a selective P2X_7_ receptor inhibitor or knockdown of the P2X_7_ receptor with siRNA, reduced the apoptosis of PTCs. The use of recombinant alkaline phosphatase has been tested both experimentally (153) and clinically (40) in sepsis-associated AKI. It is believed that the mechanism of action may involve dephosphorylation of LPS for reduced TLR activation (154) and of ATP for conversion to the anti-inflammatory adenosine (155). Binding of adenosine or its synthetic analogs to adenosine receptors protected mice from IRI in an IL-11–dependent manner (156). Adenosine was also shown to induce immune tolerance through dendritic cells (157) and T-regulatory cells (Tregs) (158). Conversely, inhibition of adenosine kinase with a small molecule (ABT-702) to prevent the conversion of adenosine to ATP was protective in cisplatin nephrotoxicity (159). Paradoxically, extracellular nucleotides including ATP released from dying cells were also shown to promote wound repair in renal tubular injury (160).

#### Secreted and Extracellular Alarmins

Blocking of glycans with doxycycline, a broad-spectrum matrix metalloprotease inhibitor, was shown to restrict the secretion of pro-inflammatory cytokines in cisplatin and IRI-induced AKI (161, 162). Heparanase inhibitors such as PG545 was found protective in experimental ischemic IRI (63) and is currently in clinical testing. Mice receiving a diet containing 4-methylumbelliferone, a potent hyaluronic acid synthesis inhibitor, resulted in attenuation of AKI (66). Pharmacological treatment with a zinc chelator, dithizone, resulted in depletion of Paneth cell granules in adult mice (163) and rats (164). These mice exhibited less leukocyte infiltration, pro-inflammatory cytokine generation, and reduced epithelial necrosis and apoptosis. In contrast, studies have also indicated that a chronic loss of Paneth cell α-defensin expression could also skew toward a more pro-inflammatory phenotype (165). These opposing outcomes warrant additional mechanistic studies to fully understand the role of defensins in AKI.

### Direct Application of Alarmins

#### Nuclear Alarmins

In contrast to the pro-inflammatory reports of IL-33, evidence also suggests that IL-33 is a potential mediator of type 2 immunity and a regulator of the protective immune response (166, 167). We identified that ST2, the receptor for IL-33, is regulated by IL-2 (168) and is expressed on a major subset of Tregs (169). Based on our data that IL-2 and IL-33 by themselves increased Tregs and partially protected from IRI and that these cytokines synergize to completely protect from AKI, we generated a novel hybrid cytokine (termed IL233) bearing activities of IL-2 and IL-33 in a single molecule (169). Treatment with IL233 robustly increased Tregs and the group 2 innate lymphoid cells (ILC2) and strongly protected kidneys from IRI, as well as cisplatin- and doxorubicin-induced nephrotoxic injuries (169, 170). A similar strategy of using exogenous IL-33 alone was demonstrated to increase ILC2 to protect from IRI in T cell–independent manner (171). Interestingly, reduction or depletion of ILC2 did not affect the severity of IRI in a mouse model, suggesting that ILC2 may be redundant for IRI (172), despite the finding that the adoptive transfer of *ex vivo*–expanded ILC2 was protective in murine IRI (169).

#### Cytoplasmic Alarmins

Preconditioning the mice with rHMGB1 prior to IRI protects the kidney against IRI was indicated by low serum creatinine, tubular damage, and tubulointerstitial neutrophil and macrophage infiltration (173). Pretreatment with rHMGB1 resulted in the upregulation of Siglec-G, which in turn negatively regulated HMGB1-mediated TLR4 pathway activation. This indicated significant protection from renal IRI from the activation of TLR4-dependent inflammatory response. It was also observed that lentivirus-mediated renal overexpression of HSP27 prevented the loss of renal function and decreased necrosis, inflammation, apoptosis, and F-actin cytoskeleton after IRI injury in mice (174). In a retrospective observational study, it was found that the intraoperative administration of haptoglobin administration was independently associated with a lower risk of AKI incidence after cardiovascular surgery (175).

Studies in 1989 identified heme oxygenase 1 (HO-1) as a protein induced in hypoxic cells. Protective responses of HO have been confirmed in various AKI studies (176). HO-1 participates in the dissipation of heme, thereby protecting the kidneys from inflammation and cellular damage. Induction of HO-1 and ferritin in the kidney protects against heme-induced kidney injury (177). HO-1 induction by granulocyte colony-stimulating factor has been shown to protect against AKI both *in vivo* and *in vitro* (178). Adiponectin, a cytokine produced from white fat, induces HO-1 in renal epithelial cells *in vitro* and prevents AKI following IRI (179). Along with heme, ferrous iron (Fe) that is found in heme also correlated with AKI (180). Administration of the iron-regulating hormone hepcidin reduced inflammation and decreased oxidative stress in mouse models of AKI (181). Further, the administration of a furin inhibitor to induce high levels of hepcidin also reduced AKI in mouse models (182).

#### Extracellular Matrix and Cell-Surface Alarmins

The use of extracellular matrix-associated alarmins for protection in AKI is largely understudied but is gaining attraction. In an interesting study (183), an HA-curcumin prodrug targeting the HA receptor-CD44 could assist in epithelial cell survival from oxidative stress during AKI. CRAMP-deficient (Cnlp^−/−^) mice exhibited exacerbated renal dysfunction accompanied by aggravated inflammatory response and apoptosis (72). Exogenous treatment with CRAMP markedly attenuated AKI accompanied by reduced NLRP3 orchestrated inflammatory response and apoptosis. In LPS-induced inflammatory settings, it was observed that overexpression of TIMP-2, a major diagnostic marker of AKI, significantly attenuated the production of nitric oxide, TNF-α, IL-1β, and ROS with increased production of anti-inflammatory cytokine (IL-10) (184). Future studies on the use of TIMP-2 are likely to produce interesting results.

### Implications of Alarmins in Repair Post-AKI

The renoprotective role of alarmins also suggests their potential in repair after renal injury. Stem cells play an important role in tissue homeostasis, as well as tissue repair following injury (185). Researchers have used exogenous stem cells to improve tissue regeneration using a variety of approaches. However, still, there is a very limited clinical success than anticipated especially for solid organ injuries (185). Alternatively, harnessing the endogenous tissue-resident stem cells for mediating repair could be promising. In a breakthrough study in 1970, it was observed that priming injury at a distant site at the time of, or before the second trauma, resulted in accelerated repair (186, 187). In a recent study, Lee et al. (188) have used the alarmin, HMGB1, to accelerate repair using a bone fracture model. Exogenous treatment with HMGB1 accelerated facture healing through the formation of heterodimer complex between HMGB1 and chemokine, CXCL12 (stromal cell–derived factor1), which then signals through CXCR4 receptor (188). Because remote ischemic preconditioning was accompanied by an upregulation of HMGB1 (189), preconditioning with recombinant HMGB1 was tested and found to be protective in AKI (173). Such an approach may as well be investigated to promote repair in AKI.

Heat shock proteins, although identified as biomarkers for AKI, are now being investigated for their beneficial role in AKI. HSP73 and HSP90 were found to be induced in the injured PTC and loop of Henle early on after injury and then were upregulated again in the regenerating cells, suggesting these HSPs may participate in repair post-IRI, and may be exploited in future studies (94). HSP70 was shown to interact with cytoskeletal elements during the restoration of the cytoskeletal structure and polarity of proximal tubules after ischemic injury, indicating the role of HSP70 in renal repair (190). An interesting concept is that T-cell reactivity to HSP may induce tolerogenic responses, which may be beneficial for the resolution of inflammatory diseases (21, 191, 192). Indeed, a recent study showed that, in a murine model of IRI, heat preconditioning induced the release of HSP-70, which in turn promoted the expansion of Tregs that was renoprotective (193, 194).

A reparative role of Tregs in AKI was initially shown in murine IRI through depletion studies (195). Recently, we demonstrated that treatment with the fusion protein IL233 utilizes the synergy of IL-2 with the IL-33 alarmin in protection when administered after the onset of injury (169). IL233 treatment, initiated 2 weeks after renal injury, induced near-complete restoration of renal structure and function (170). IL233 treatment invoked the proliferation and renal recruitment of Tregs and ILC2s. Antibody-mediated depletion of these cells ameliorated the restoration of renal injury. Further, mobilization of these cells near the site of injury promoted the recruitment of progenitor cells in the kidneys. It remains to be evaluated whether this may be either a direct effect of these cells or through inducing an anti-inflammatory milieu, which may be conducive for progenitor cells to promote regenerative responses. Treatment with IL233 after the onset of lupus nephritis and diabetic nephropathy in animal models also induced persistent remission, suggestive of a reparative role of IL-33 alarmin in chronic renal injury (170, 196, 197). Current studies in our group are addressing the role of the IL-33/ST2 and IL233 in the repair of renal injury in both an immune-dependent and independent manner.

## Conclusion

The immunoregulatory potential of alarmins, as well as their predictive value as a biomarker in a host of disease conditions, renders the study of alarmins beneficial for clinical applications. Despite all the advances in the understating of the pathophysiology of kidney diseases, the dearth of treatment strategies for AKI remains a major unmet clinical need. Novel therapeutic options or perhaps a combination of those in a concerted manner is required to solve this problem. Exploring the role of alarmins as diagnostic markers, immunomodulators, and harbingers of repair could be one of the strategies that may lead to therapy of AKI.

## Author Contributions

RS conceived the idea and performed the final revision. VS performed the bulk of literature search in collaboration with RV and MD. RS, VS, RV, and MD co-wrote the manuscript. All authors contributed to the article and approved the submitted version.

## Conflict of Interest

The authors declare that the research was conducted in the absence of any commercial or financial relationships that could be construed as a potential conflict of interest.
